# Comparative analysis of adaptive and general labeling methods for soybean leaf detection

**DOI:** 10.3389/fpls.2025.1582303

**Published:** 2025-06-16

**Authors:** Yuseok Jeong, Song Lim Kim, Thanh Tuan Thai, Anh Tuan Le, Chaewon Lee, Hyo Jun Bae, Inchan Choi, Sheikh Mansoor, Yong Suk Chung, Kyung-Hwan Kim

**Affiliations:** ^1^ Department of Agricultural Engineering, National Institute of Agricultural Sciences, Rural Development Administration (RDA), Jeonju, Republic of Korea; ^2^ School of Computer Information and Communication Engineering, Kunsan National University, Gunsan, Republic of Korea; ^3^ Digital Breeding Convergence Division, National Institute of Agricultural Sciences, Rural Development Administration (RDA), Jeonju, Republic of Korea; ^4^ Communication Multimedia Laboratory, University of Information Technology, Ho Chi Minh, Vietnam; ^5^ Vietnam National University, Ho Chi Minh, Vietnam; ^6^ Department of Plant Resources and Environment, Jeju National University, Jeju, Republic of Korea; ^7^ Faculty of Biology—Biotechnology, University of Science, Ho Chi Minh, Vietnam; ^8^ Crop Production and Physiology Research Division, National Institute of Crop and Food Science, Rural Development Administration (RDA), Wanju-Gun, Republic of Korea; ^9^ Supercomputing Center, National Institute of Agricultural Sciences, Rural Development Administration (RDA), Jeonju, Jeonju, Republic of Korea

**Keywords:** deep learning, image analysis, object detection, phenotyping, crop management

## Abstract

Soybeans are important due to their nutritional benefits, economic role, agricultural contributions, and various industrial applications. Effective leaf detection plays a crucial role in analyzing soybean growth within precision agriculture. This study examines the influence of different labeling methods on the efficiency of artificial intelligence (AI) based soybean leaf detection. We compare a traditional general labeling technique against a new context-aware method that utilizes information about leaf length and bottom extremities. Both approaches were employed to train a YOLOv5L deep learning model using high-resolution soybean imagery. Results show that the general labeling method excelled with soybean varieties that have wider internodes and distinctly separated leaves. In contrast, the context-aware labeling method outperformed the general approach for medium soybean varieties characterized by narrower internodes and overlapping leaves. By optimizing labeling strategies, the accuracy and efficiency of AI-based soybean growth analysis can be significantly improved, particularly in high-throughput phenotyping systems. Ultimately, the findings suggest that a thoughtful approach to labeling can enhance agricultural management practices, contributing to better crop monitoring and improved yields.

## Introduction

Soybean (Glycine max) is a globally important legume, serving critical roles in the food, feed, and biofuel sectors (Mansoor et al., 2024). Soybeans hold significant importance in global agriculture and nutrition due to their diverse applications and nutritional benefits. Rich in protein, soybeans are a critical food source, particularly in vegetarian and vegan diets, providing all essential amino acids necessary for human health (Messina, 1999; Kastner et al., 2021; Nair et al., 2023). Economically, soybeans are an essential crop for numerous countries, particularly the United States, Brazil, and Argentina, which are the leading producers. Soybeans’ versatility extends beyond direct consumption; they are transformed into various products such as tofu, soy milk, and soy sauce, and are also integral to animal feed, bolstering the meat and dairy industries (Klein and Luna, 2021; Toloi et al., 2021; Hamza et al., 2024). In agriculture, soybeans significantly contribute to sustainable farming practices. As legumes, they possess the unique ability to fix nitrogen in the soil through symbiotic relationships with bacteria. This natural process reduces the need for synthetic nitrogen fertilizers, improving soil fertility and health. Consequently, incorporating soybeans into crop rotations can enhance the productivity of other crops and foster more sustainable farming systems (Liu et al., 2020; Goyal et al., 2021).

Image analysis and artificial intelligence (AI) are revolutionizing soybean agriculture by enhancing crop management, improving yield prediction, and aiding in disease detection. Advanced image processing techniques, combined with machine learning algorithms, provide precise and timely information that helps farmers make informed decisions, ultimately leading to more efficient and sustainable farming practices (Redhu et al., 2022; Mesías-Ruiz et al., 2023; Sheikh et al., 2023; Jafar et al., 2024; Kashyap et al., 2024; Mansoor et al., 2024). High-resolution satellite imagery and drones equipped with multispectral cameras are increasingly used for monitoring soybean fields. These tools capture detailed images that can be analyzed to assess plant health, identify nutrient deficiencies, and detect water stress (Mansoor and Chung, 2024; Zhang et al., 2020; Olson and Anderson, 2021; Karunathilake et al., 2023). Accurate yield prediction is crucial for planning and market strategies. AI models, trained on historical yield data and environmental factors, can predict soybean yields with high accuracy. These models incorporate various data sources, including weather patterns, soil conditions, and planting density, to forecast yields (Han et al., 2020; Morales and Villalobos, 2023; von Bloh et al., 2023).

Optimizing yield and resource management in soybean production necessitates accurate monitoring and analysis of growth stages. Image analysis techniques have gained traction in soybean research, with applications such as flower and pod detection in field videos using deep learning models (Gaso et al., 2021; Vogel et al., 2021; Shammi et al., 2024). However, studies focusing on the foliate sequence, the order and timing of leaf development, remain scarce. The foliate sequence in soybeans progresses from the coleoptile to the fifth compound leaf, with the timing of leaf emergence serving as a valuable indicator of overall crop growth.

The performance of artificial intelligence (AI) models is highly dependent on the quality and relevance of input data. Effective labeling methods, tailored to the specific AI model and its purpose, are crucial for achieving optimal performance (Araújo et al., 2021; Jagatheesaperumal et al., 2021; Liang et al., 2022). Common labeling techniques include square or polygon methods for object detection, segmentation methods for object segmentation, and image-to-text or text-to-image methods for generative models (Baraheem et al., 2023). Selecting the appropriate labeling approach is essential for ensuring desirable model performance. Accurately marking the boundaries of leaves is vital, ensuring precise spatial information for models (Guo et al., 2024). For tasks like distinguishing leaves within plant canopies, instance segmentation is essential, delineating each leaf distinctly for accurate identification (Afzaal et al., 2021; Ku et al., 2023; Kwon et al., 2024). Annotating leaf landmarks supports detailed analysis like disease detection or growth stage classification (Arya et al., 2022). Adding metadata such as leaf age or health enriches datasets, aiding model generalization (Rayhana et al., 2023). Consistent labeling across datasets and annotators is critical for effective model training (Waldamichael et al., 2022).

Motivated by the above-mentioned considerations, this study investigates the application of AI for soybean leaf detection with the aim of enhancing soybean growth analysis. To achieve this, we propose a new labeling method that incorporates the growth characteristics of soybeans. This method will be compared against a General labeling method approach to evaluate its impact on the accuracy of detecting soybean leaves relative to the plant’s growth stage.

In recent years, various machine learning approaches have been explored for plant phenotyping and leaf detection. YOLO-based models have been widely applied due to their real-time detection capabilities and high performance in identifying plant organs such as fruits and leaves in crops like tomatoes and grapes (Afzaal et al., 2021; Ku et al., 2023). However, these models rely on bounding boxes, which often struggle in scenarios involving dense foliage or overlapping leaves.

To overcome these limitations, instance segmentation models like Mask R-CNN (He et al., 2017) and transformer-based object detectors such as DETR (Carion et al., 2020) and DINO (Zhang et al., 2023) have emerged. These models can distinguish object boundaries more precisely by producing pixel-level segmentation, albeit at higher computational costs.

Recent studies on plants such as Arabidopsis thaliana, strawberries, and citrus fruits have leveraged side-view high-throughput phenotyping systems to monitor growth traits over time (Sampaio et al., 2021; Xiong et al., 2021; Kwon et al., 2024). Moreover, the labeling process itself has been recognized as a significant factor influencing model performance, particularly in occluded or complex structures (Guo et al., 2024).

Despite these advancements, few studies have systematically examined how labeling strategies affect object detection performance across varying plant architectures. Therefore, this study focuses on comparing general and context-aware labeling methods for soybean leaves, considering different internode lengths, to enhance detection accuracy in high-throughput phenotyping settings.

## Materials and methods

### Data collection

### Soybean varieties and selection criteria

Three soybean varieties were selected based on their internode length, a key indicator of growth form. Hefeng, Dawon, and Hannam were chosen to represent long, medium, and short internode varieties, respectively. This selection strategy allows us to investigate the impact of plant architecture on leaf detection performance.

### Image acquisition system and data description

Soybean image data was obtained from the Crop phenomics research center of the Rural Development Administration. The center utilizes a high-throughput phenotyping system where soybean plants are placed on a conveyor belt and transported through a filming area equipped with a high-resolution camera (4,384 x 6,576 pixels). This system captures images from three different three lateral angles (0°, 120°, and 240°) for each plant, resulting in a total of six images per plant (two per day, once in the morning and once in the afternoon). The image filenames encode detailed information including the crop’s serial number (B_06_35), shooting date (2018-09-11), shooting time (14:55:07), and image equipment details (VIS_SV0). Data collection spanned a period of 22 days, from August 21st, 2018, to September 11th, 2018. A total of 5,547 images were collected, comprising 1,851 images for each variety (short, medium, and long internode). This balanced distribution across varieties ensures a statistically robust analysis of the proposed labeling method.

### Data preprocessing

#### Region of interest extraction

**Figure 1:**
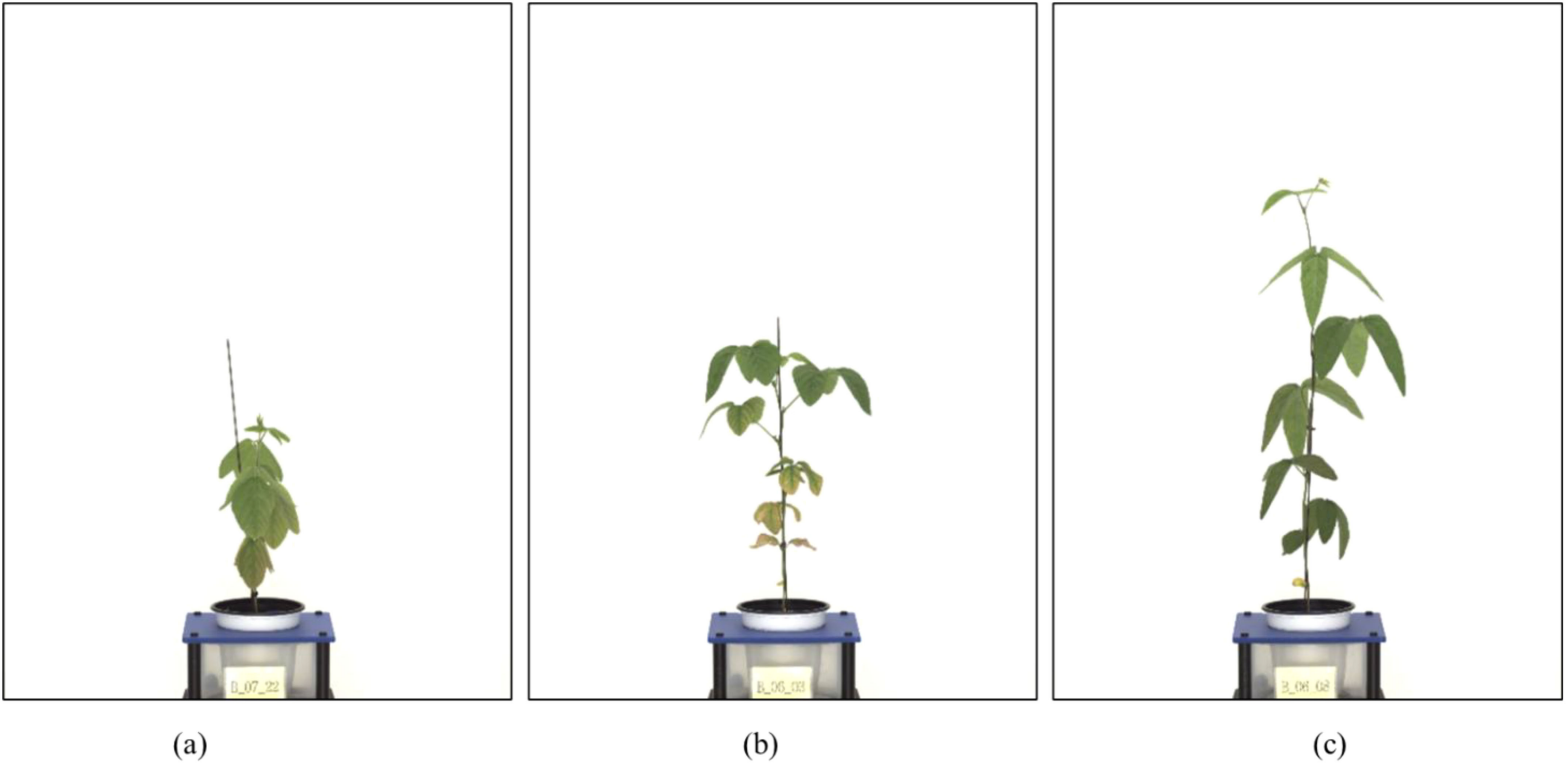
Examples of preprocessed soybean images (ROI extraction results). **(a)** Short-internode variety (Hannam), **(b)** Medium-internode variety (Dawon), **(c)** Long-internode variety (Hefeng). Each image illustrates the result of extracting a region of interest (ROI) by removing the background and focusing on the central part of the plant.

The original images (4,384 x 6,576 pixels) included significant background components unrelated to the plant structure. Therefore, we implemented a custom image preprocessing pipeline in Python, which identified the central vertical axis of each plant and extracted a rectangular region of interest (ROI) around it. The segmentation was based on simple pixel intensity thresholds and geometric heuristics that isolated the vertical green structure (stem and leaves) from the white background. The extracted ROI images were resized to 2,401 × 2,951 pixels, preserving key spatial features while reducing computational overhead during model training. **Figure 1** illustrates representative examples of the extracted ROI for each soybean variety.

### Data labeling

**Figure 2:**
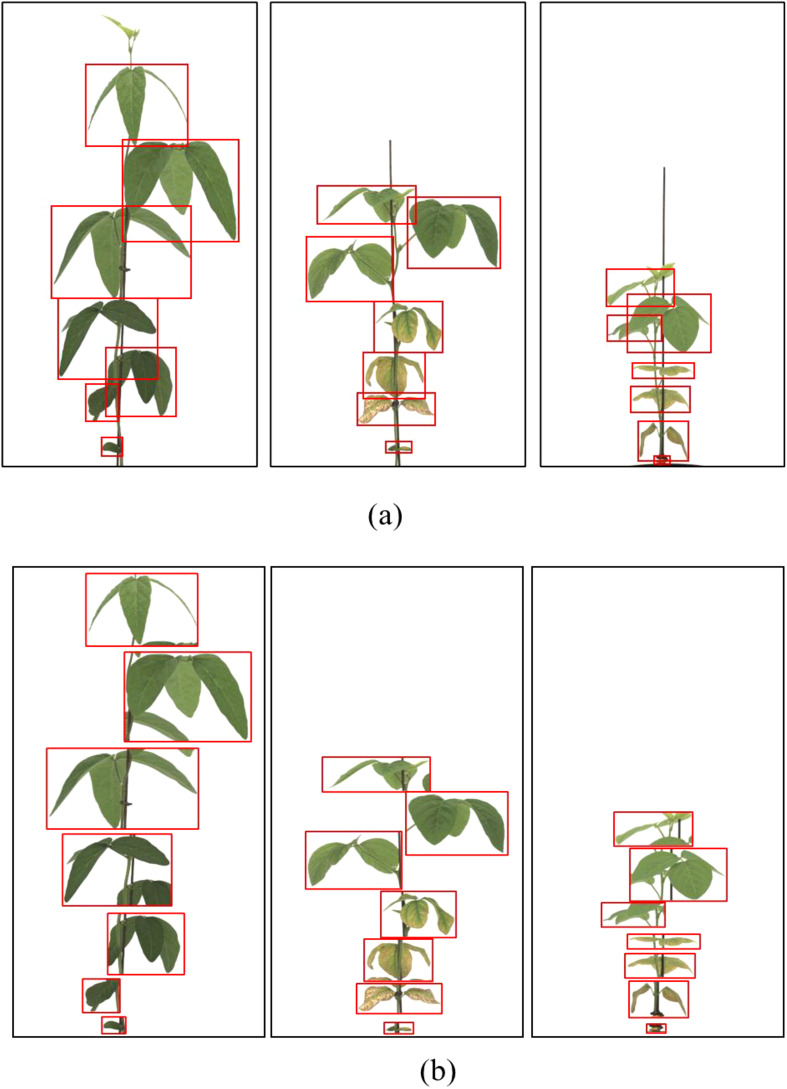
General labeling method **(a)** Bounding box annotation applied to clusters of overlapping leaves, **(b)** Zoomed-in view of labeled regions. Leaves are annotated collectively when boundaries are unclear, which is effective for simple plant structures but less accurate in overlapping scenarios.

Data labeling was performed using the coordinate data generation program LabelImg (MIT). Two labeling strategies were compared. General Labeling Method: This approach involved drawing bounding boxes around clusters of visible leaves, treating overlapping leaves as a single object when boundaries were ambiguous. This is a commonly used strategy in many agricultural datasets, especially for high-throughput or large-scale image collections, due to its efficiency (**Figure 2**).

**Figure 3:**
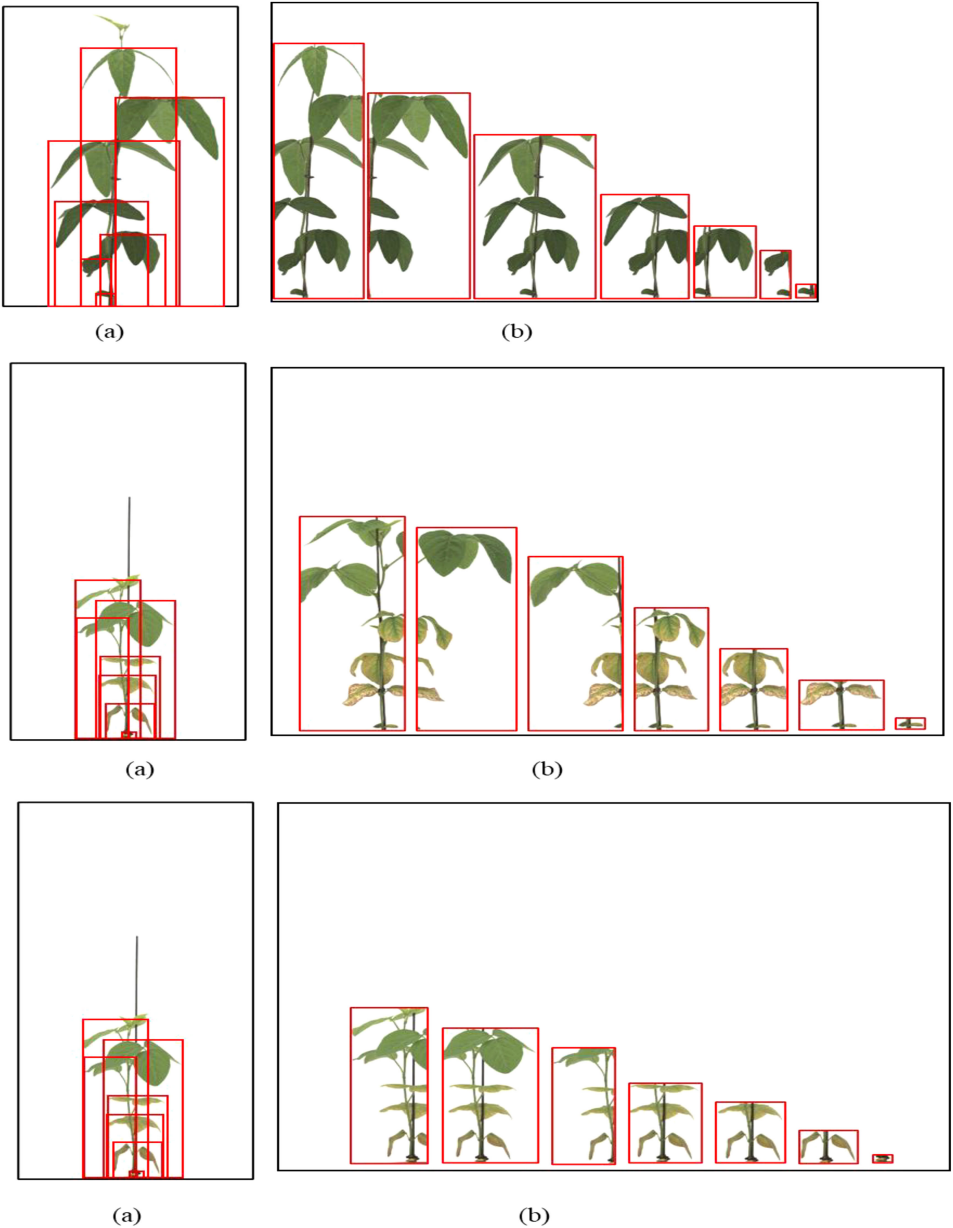
Proposed context-aware labeling method. **(a)** Individual leaf annotations incorporating lower reference points, **(b)** Enlarged view showing contextual labeling of overlapping leaves. Each leaf is annotated separately, considering emergence angle and attachment point, which allows for better differentiation in complex growth stages.

Context-aware labeling method (proposed): In this method, annotators labeled each visible leaf or partial leaf individually, incorporating additional contextual cues. The vertical extent (height) of each leaf from petiole to tip. The bottom reference point (where the petiole attaches to the stem) was used to guide separation between overlapping leaves. Annotators were instructed to consider the angle of emergence, treating leaves as fully developed only if the trifoliate had horizontally unfolded. This labeling style was designed to disambiguate leaf boundaries in overlapping settings and align with known biological development stages (Szczerba et al., 2021; Vogel et al., 2021; Kaspar, 2022) (**Figure 3**).

The labeling process required approximately 370 hours in total, emphasizing the need for future integration of semi-automated annotation systems or active learning-based labeling suggestions.

### Artificial intelligence model for leaf detection

YOLOv5L was used for the model for detecting leaf objects in image data of soybean crops. YOLO divides the input image data into grid cells and detects objects when the center of an object falls within one of these cells. Each grid cell predicts bounding boxes and a confidence score for each bounding box. The confidence score indicates the model’s confidence in detection, showing both the probability that a bounding box contains an object and the accuracy of the predicted bounding box. The calculation formula for confidence is given by Equations 1, 2.


(1) $$Confidence\;Score=\text{Pr}\left(Object\right)\times IoU_{pred}^{truth}$$

(2) $$IoU_{pred}^{truth}=\frac{B_{truth}\cap^{}B_{pred}}{B_{truth}\cup^{}B_{pred}}\;$$

Additionally, when each grid cell contains an object, the model also computes the probability for each class. Subsequently, the process of detecting objects in the image data involves multiplying the confidence score for each bounding box by the probability for each class, and the calculation formula is as shown in Equation 3. represents the probability that the object contained in the grid cell is class i.


(3) $$\mathrm{Pr}\left(Class_{i}|Object\right)\times \mathrm{Pr}\left(Object\right)\times IoU_{pred}^{truth}=\text{Pr}\left(Class_{i}\right)\times IoU_{pred}^{truth}$$

**Figure 4:**
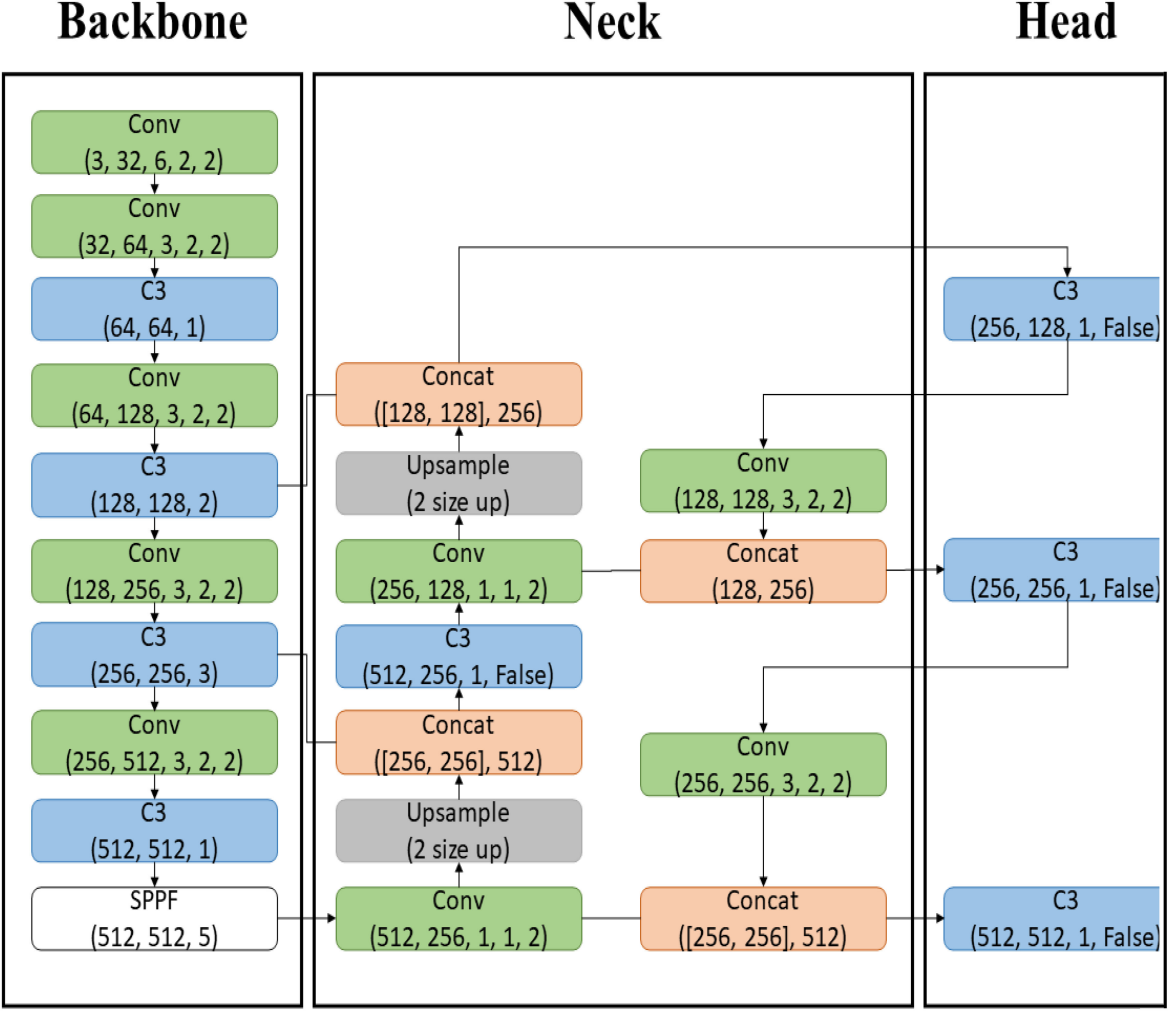
YOLOv5L model structure. The Backbone, implemented in PyTorch with CSP-Darknet, transforms input images into feature maps through C3 convolutional operations. The Neck upsamples and concatenates these feature maps to enhance feature extraction, while the Head stage performs object localization and classification, enabling effective multi-object detection from a single image.

The most significant change in YOLOv5 is the implementation of the Backbone, previously in C language, now in PyTorch, utilizing CSP-Darknet. The Backbone functions to transform input image data into feature maps through convolutional (Conv) operations, specifically C3 operations. The Neck upsamples the feature maps to increase their size and concatenates them with other feature maps, harmonizing the extracted features from the Backbone. The Head stage performs localization and object classification. With multiple feature maps in the Neck, the model can effectively detect multiple objects from a single image data. The structure of YOLOv5L used in this paper is depicted in **Figure 4**.

### Model training

To obtain an optimal artificial intelligence model, various methods, models, and parameter settings were explored. This process requires substantial time and access to the latest high-capacity equipment. However, due to modifications in simple labeling methods, the learning environment was limited for optimization through performance comparison. The learning parameters were kept consistent: Epoch 300, Batch Size 2, Image Size 1920, and Optimizer Adam. The learning environment consisted of an Intel Core i7-11700K processor, 128GB RAM, and an NVIDIA GeForce RTX 3080 TI 12GB GPU. The study used data from three soybean varieties with different internode lengths: Hannam (short internodes), Dawon (medium internodes), and Hefeng (long internodes), applying both the general labeling method and the proposed labeling method. Specifically, 1,473 images of the short variety (Hannam), 1,473 images of the medium variety (Dawon), and 1,467 images of the variety with long internodes (Hefeng) were used as learning data.

### Model performance evaluation method

The performance of the object detection artificial intelligence model is evaluated using a confusion matrix that compares the predicted results with the actual values. In the confusion matrix, True Positive (TP) represents the correctly classified instances of the category of interest, while False Positive (FP) represents instances incorrectly classified as the category of interest. True Negative (TN) indicates instances correctly identified as not belonging to the category of interest, and False Negative (FN) denotes instances that were incorrectly classified as not being the category of interest. Using the values from the confusion matrix, three key measures—Accuracy, Precision, and Recall—can be calculated.

Accuracy measures the overall correctness of the model’s predictions by evaluating how well it predicts both True and False values (Equation 4). Precision measures the accuracy of the model’s positive predictions, defined as the ratio of true positive predictions to the total number of instances predicted as positive (Equation 5). Recall, also known as sensitivity, is the ratio of correctly predicted positive instances to the actual total number of positive instances, indicating how well the model identifies the category of interest (Equation 6).


(4) $$Accuracy=\;\frac{TP+TN}{TP+FN+FP+TN}$$

(5) $$Precision=\;\frac{TP}{TP+FP}$$

(6) $$Recall=\;\frac{TP}{TP+FN}$$

## Results

### Performance evaluation based on labeling method and performance evaluation of variety with short internodes (Hannam)

**Figure 5:**
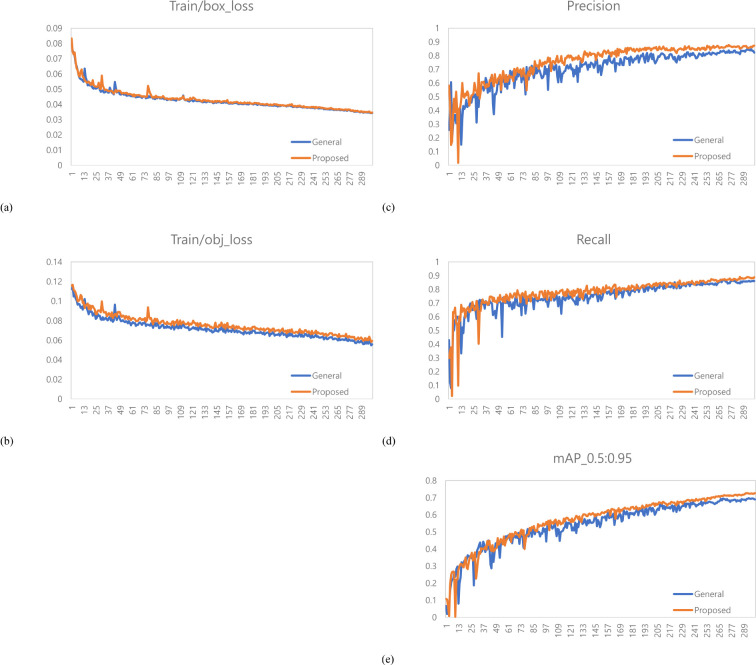
Training results graph for Hannam. **(a)** Train box loss comparison graph, **(b)** Train object loss comparison graph, **(c)** Precision comparison graph, **(d)** Recall comparison graph, **(e)** mAP_0.5:0.95 Graph.

A total of 1,473 images of the variety with short internodes (Hannam) were used for learning data. The learning environment was controlled to compare performance based on changes in the labeling method. The result graph presents data up to Epoch 300 (**Figure 5**).

A total of 378 images were used as validation data. **Table 1** compares the performance evaluation of the general labeling method and the proposed labeling method for varieties with short internodes. The general labeling model achieved a Precision of 89.9%, Recall of 69.8%, Accuracy of 85.9%, F1 score of 78.6%, and an mAP_0.5:0.95 score of 69%. The proposed labeling model achieved a Precision of 93.3%, Recall of 74.5%, Accuracy of 88.5%, F1 score of 0.82.9%, and an mAP_0.5:0.95 score of 72.6%. For varieties with short internodes, the proposed labeling method showed improvements with increases of 3.4% in Precision, 4.7% in Recall, 2.6% in Accuracy, 4.2% in F1 score, and 3.6% in the mAP_0.5:0.95.

**Table 1:** Comprehensive comparison table of performance evaluation for variety with short internodes.

Style	Labels	TP	FN	FP	TN	Precision	Recall	Accuracy	F1	mAP
General	1633	1142	492	128	2646	89.9%	69.8%	85.9%	78.6%	69.0%
Proposed	1633	1209	412	86	2646	93.3%	74.5%	88.5%	82.9%	72.6%

**Figure 6:**
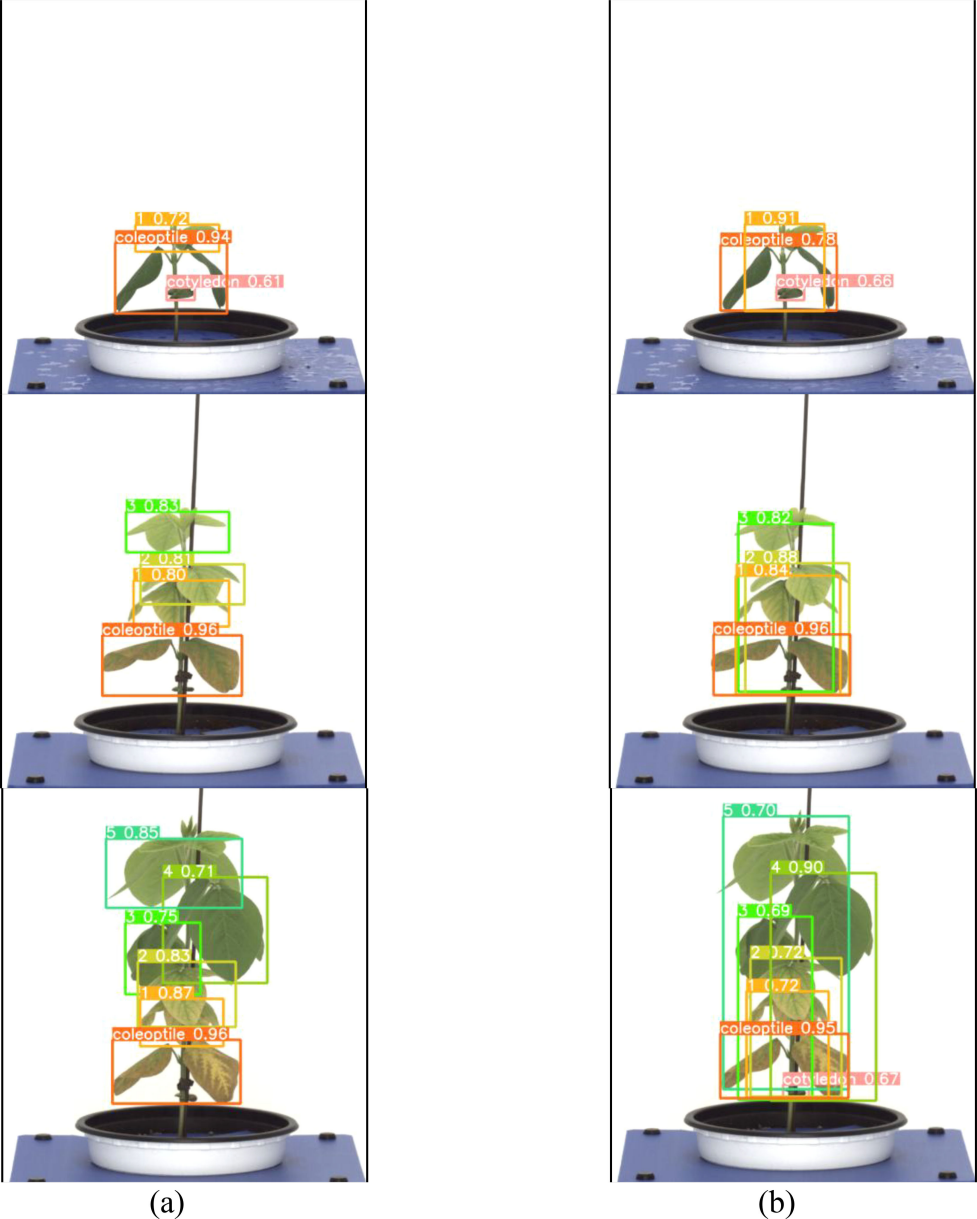
Results images for variety with short internodes. **(a)** Results with general labeling method, **(b)** Results with proposed labeling method.

Both the General labeling method and Proposed labeling methods successfully detected leaf growth stages from 1st to 5th leaf during soybean growth process (**Figure 6**).

**Figure 7:**
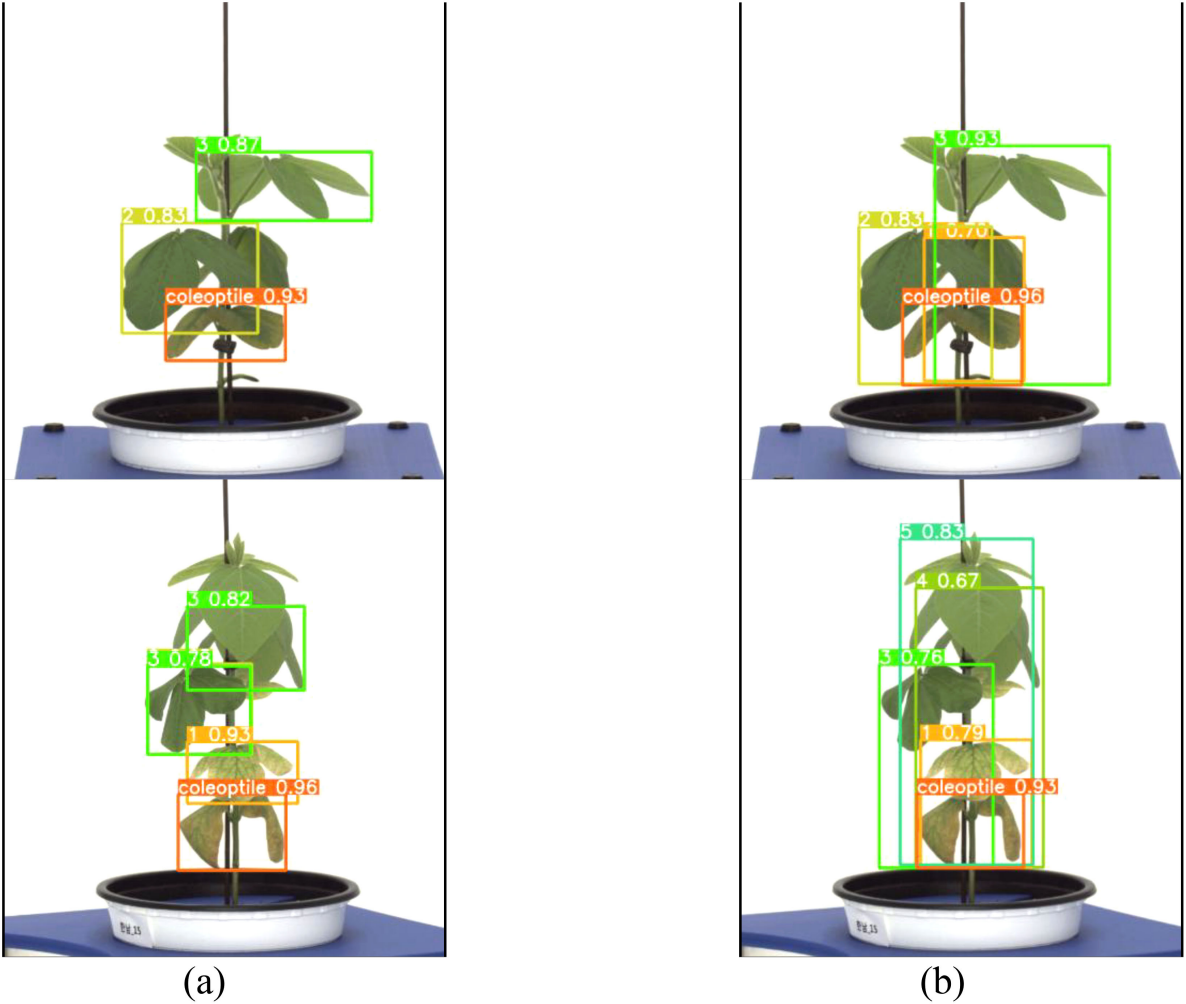
Comparison Images of Results for Variety with short internodes. **(a)** Results with General labeling Method, **(b)** Results with Proposed labeling method.

Varieties with short internodes have short internodes, resulting in many overlapping leaves. With the general labeling method (**Figure 7a**), there were instances of misdetection and non-detection in overlapping leaves. However, the YOLOv5L model trained with the proposed labeling method (**Figure 7b**) successfully detected overlapping or obscured leaves. These overlapping features contributed to the differences in detection performance.

### Performance evaluation of medium variety (Dawon)

**Figure 8:**
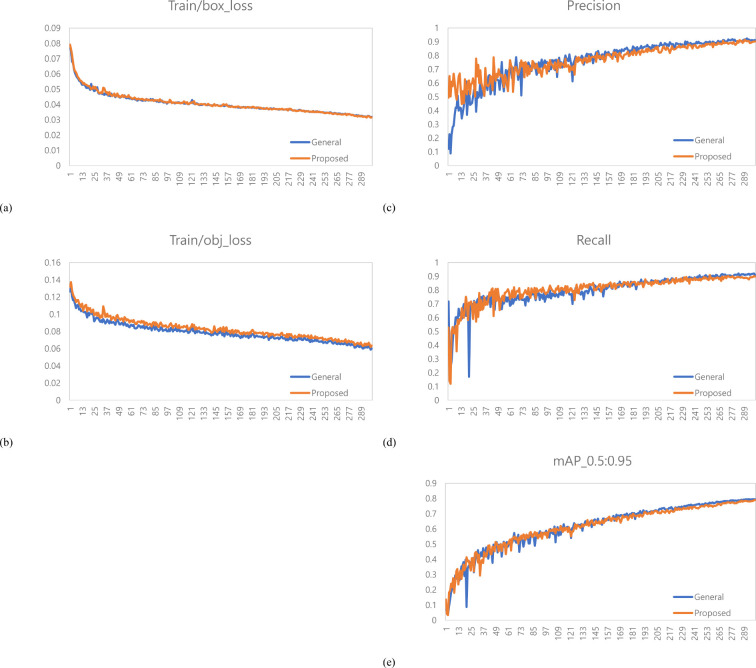
Training results graph for Dawon. **(a)** Train box loss comparison graph, **(b)** Train Object Loss Comparison Graph, **(c)** Precision Comparison Graph, **(d)** Recall Comparison Graph, **(e)** mAP_0.5:0.95 Graph.

A total of 1,473 images of varieties with intermediate growth habit (Dawon) were used as learning data. The learning environment was controlled to compare performance based on changes in the labeling method. The result graph presents data up to Epoch 300 (**Figure 8**).

A total of 378 images were used as validation data. **Table 2** compares the performance of the general labeling method and the proposed labeling method for varieties with intermediate growth habit. The general labeling model achieved a Precision of 97.2%, Recall of 81.8%, Accuracy of 91.9%, F1 score of 88.8%, and an mAP_0.5:0.95 score of 79.5%. The proposed labeling model achieved a Precision of 96.7%, Recall of 82.9%, Accuracy of 92.2%, F1 score of 89.3%, and an mAP_0.5:0.95 score of 78.8%. For varieties with intermediate growth habit, the proposed labeling method showed improved values with a Recall increase of 1.1%, an Accuracy increase of 0.3%, and an F1 score increase of 0.5%, although Precision 0.5%, mAP_0.5:0.95 was 0.7% lower compared to the general labeling method.

**Table 2:** Comprehensive comparison table of performance evaluation for medium variety.

Style	Labels	TP	FN	FP	TN	Precision	Recall	Accuracy	F1	mAP
General	1744	1427	317	41	2646	97.2%	81.8%	91.9%	88.8%	79.5%
Proposed	1744	1447	297	48	2646	96.7%	82.9%	92.2%	89.3%	78.8%

**Figure 9:**
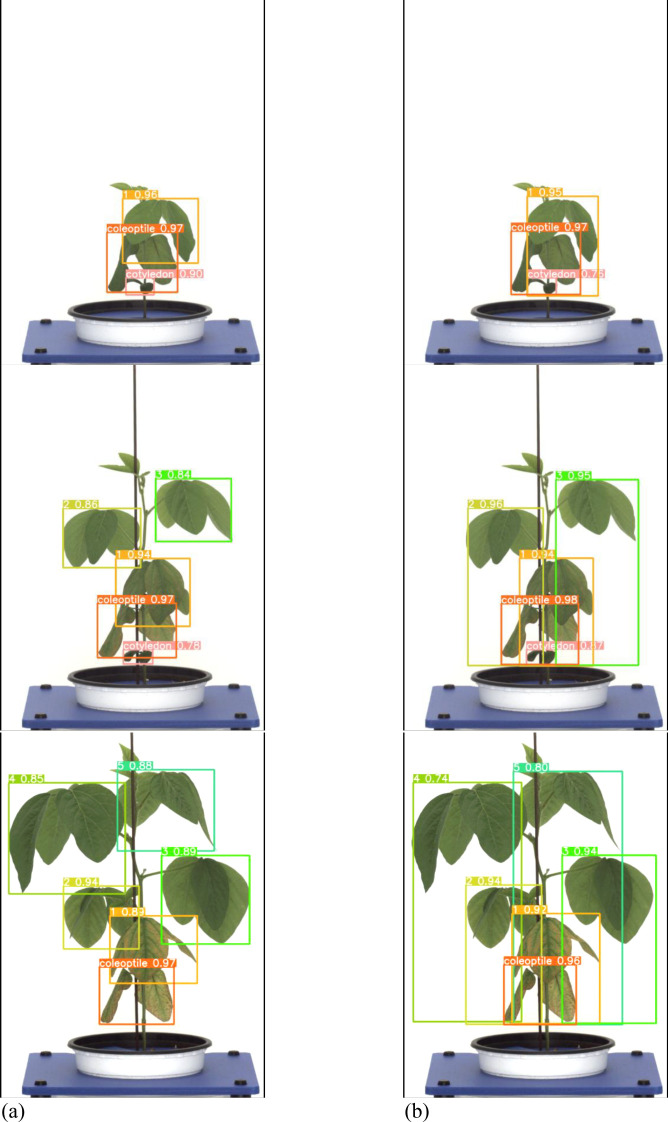
Results images for medium variety. **(a)** Results with general labeling method, **(b)** Results with proposed labeling method.

Both the general labeling method and the proposed labeling method successfully detected 1 to 5 leaves during the soybean growth process (**Figure 9**). Varieties with intermediate growth habit exhibit appropriate internode lengths and leaf overlap. There were no differences in the results between the general labeling method and the proposed labeling method.

### Performance evaluation for variety with long internodes (Hefeng)

**Figure 10:**
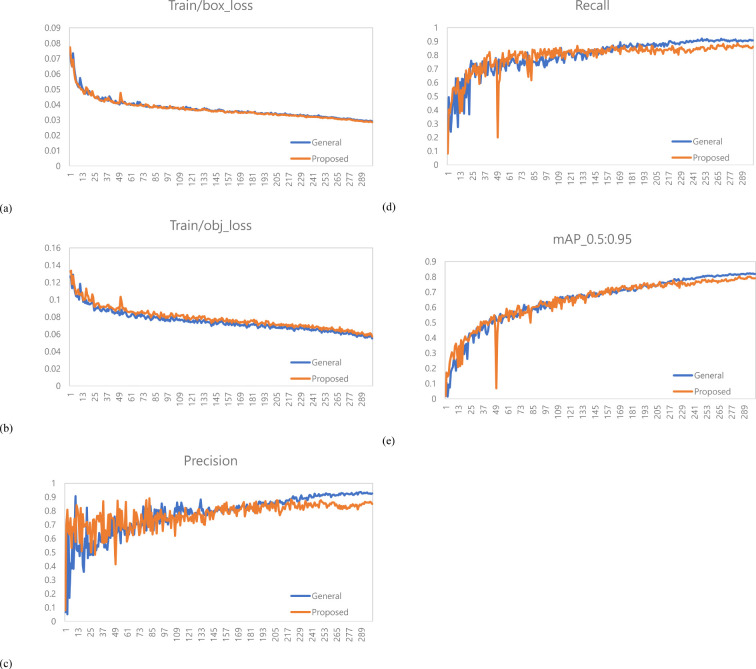
Training results graph for variety with long internodes. **(a)** Train box loss comparison graph, **(b)** Train object loss comparison graph, **(c)** Precision comparison graph, **(d)** Recall comparison graph, **(e)** mAP_0.5:0.95 Graph.

A total of 1,467 varieties with long internodes (Hefeng) were used as learning data. The learning environment was controlled to compare performance based on changes in the labeling method. The result graph presents data up to Epoch 300 (**Figure 10**).

**Figure 11:**
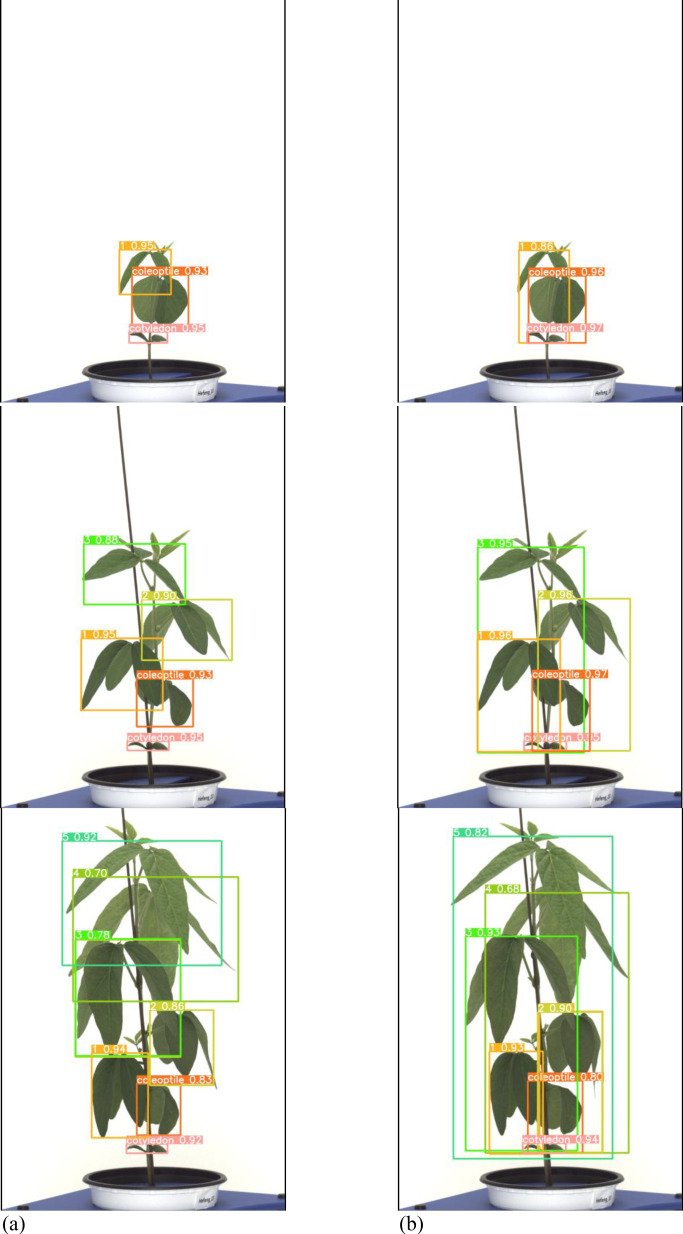
Results images for variety with long internodes. **(a)** Results with general labeling method, **(b)** Results with proposed labeling method.

A total of 378 images were used as validation data. **Table 3** presents a comparison between the general labeling method and the proposed labeling method for varieties with long internodes (**Figure 11**). The general labeling model achieved a precision of 97.0%, Recall of 88.4%, accuracy of 94.5%, F1 score of 92.5%, and an mAP_0.5:0.95 score of 81.8%. In contrast, the proposed labeling model achieved a precision of 92.6%, Recall of 85.8%, accuracy of 92.0%, F1 score of 89.0%, and an mAP_0.5:0.95 score of 78.9%. However, for varieties with long internodes, the general labeling method exhibited superior values with an increase in precision of 4.4%, recall of 2.6%, accuracy of 2.5%, F1 score of 3.5%, and an mAP_0.5:0.95 score of 3.1%.

**Table 3:** Comprehensive comparison table of performance evaluation for variety with long internodes.

Style	Labels	TP	FN	FP	TN	Precision	Recall	Accuracy	F1	mAP
General	1675	1481	194	46	2646	97.0%	88.4%	94.5%	92.5%	81.8%
Proposed	1675	1437	238	115	2646	92.6%	85.8%	92.0%	89.0%	78.9%

**Figure 12:**
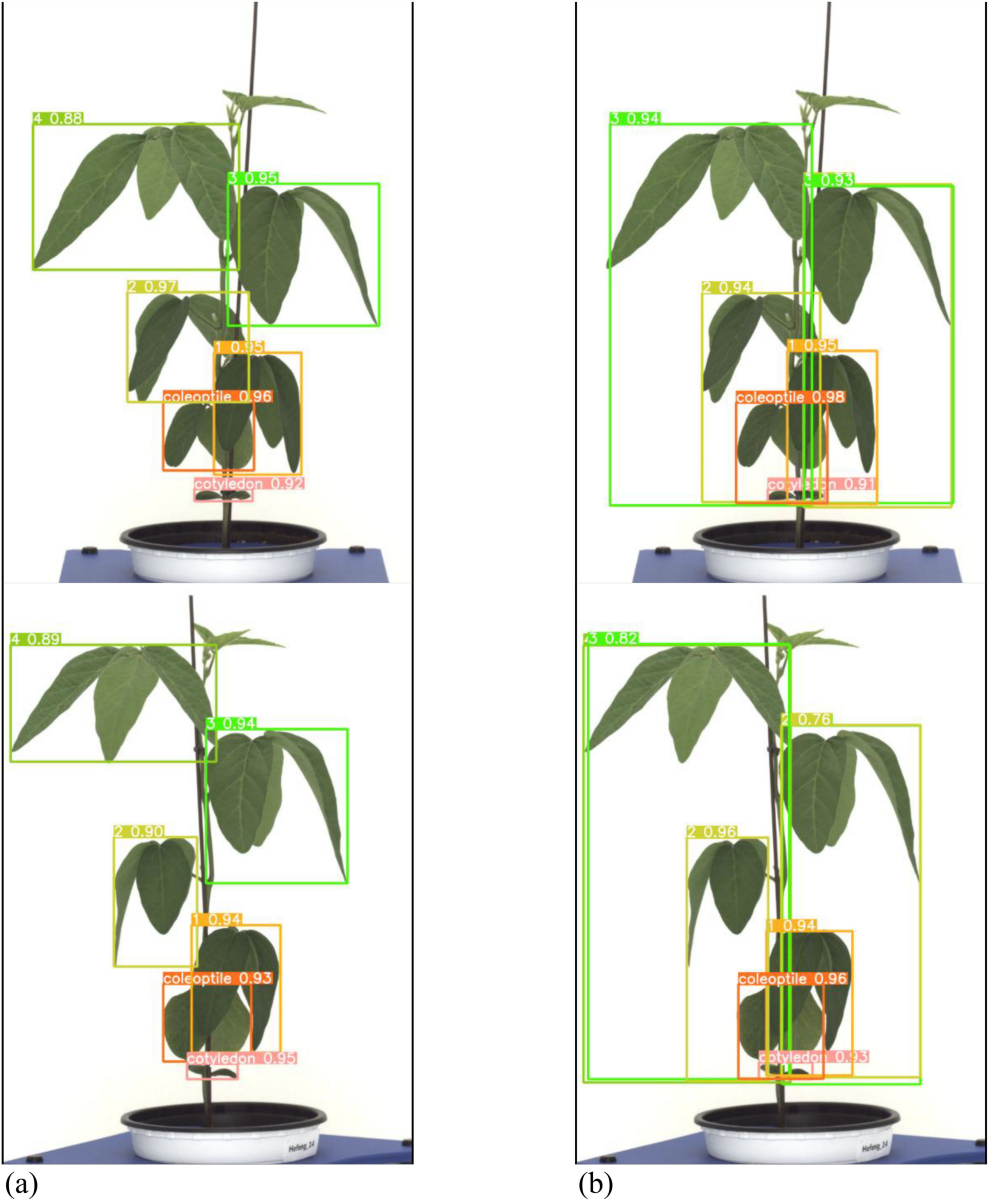
Comparison images of results for variety with long internodes. **(a)** Results with general labeling method, **(b)** Results with proposed labeling method.

Both the general labeling method and proposed labeling methods successfully detected leaf growth stages from 1st to 5th leaf during soybean growth process (**Figure 12**). In varieties with long internodes, the internodes are spacious, leading to minimal leaf overlap. With the existing method (**Figure 12a**), successful detection of all lobes was observed. However, in the proposed labeling method (**Figure 12B**), false detections were noted starting from the third lobe. This discrepancy in detection performance can be attributed to the long length of the internodes and the absence of overlapping features.

**Figure 13:**
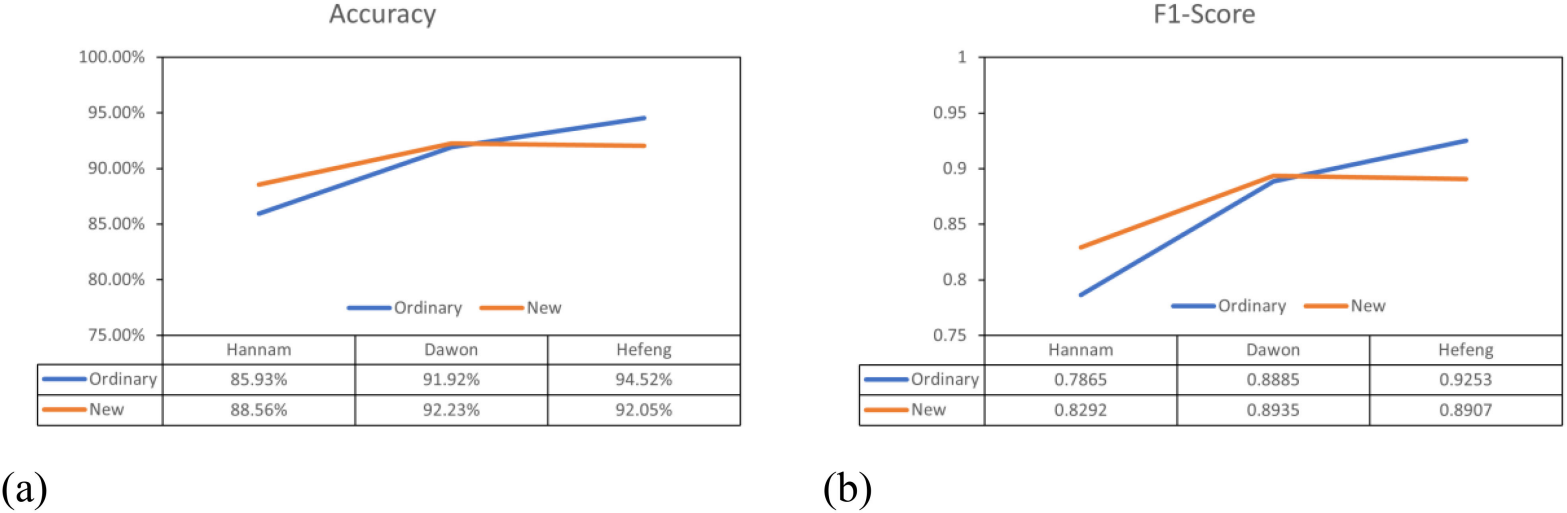
Overall performance comparison graphs. **(a)** Accuracy comparison graph, **(b)** F1-score comparison graph.

The performance of the general labeling method improved as the leaf internodes widened, reaching its peak in varieties with long internodes. Conversely, the proposed labeling method demonstrated its highest performance in mid-nodal varieties of leaves (**Figure 13**).

## Discussion

Studying crop growth patterns is an important factor in predicting crop growth status and yield. Nowadays, phenotypic research in crops using cutting-edge equipment such as hyperspectral sensors, LiDAR sensors and UAVs is becoming increasingly active (Fan et al., 2021; Omia et al., 2023; Patil et al., 2024) For soybeans, research is being conducted on disease detection through leaf analysis, bioclimatic prediction based on relative maturity groups of soybeans, and growth analysis using aerial imagery (Raza et al., 2020; Zhou et al., 2020; Radočaj et al., 2021). Soybean plant architecture, the spacing between internodes significantly affects the arrangement and visibility of leaves (Wang et al., 2021; Sreekanta et al., 2024). However, there is still a lack of research that recognizes individual bean leaves growing at each stage and analyzes the growth patterns of soybeans. In soybean growth, as the internodes widen, compound leaves exist independently and distinctly. Therefore, a general labeling method proves effective. Conversely, as the internodes narrow, compound leaves tend to overlap, suggesting that a labeling method incorporating contextual overlap would be preferable to a general labeling approach. Determining the time interval between the first and second compound leaf stages offers the potential to predict subsequent leaf emergence and identify varietal growth differences. This information can further guide the selection of suitable climates and growing conditions for specific soybean varieties.

When internodes are sufficiently spaced, each compound leaf is distinct, simplifying the labeling process (Xiong et al., 2021). However, as internodes shorten and leaves overlap, more sophisticated methods that account for overlapping contexts are required to accurately label and analyze the leaves (Sampaio et al., 2021). To assess the growth of soybean crops, it is necessary to analyze the formation and deployment of soybean leaves in order to determine the trifoliate. This is because growth analysis can be conducted based on the period of trifoliate formation. When new soybean leaves are formed, the tips of the leaves point upwards and gradually unfold sideways as they develop. In the process of soybean growth analysis, the appearance of the nth trifoliate (Vn) is considered complete when the inclination of the leaves is oriented horizontally downwards. In Kaspar, 2022 (Tong et al., 2022) the vegetative stages of soybean growth are defined by the number of nodes on the main stem with fully developed leaves beginning with the unifoliolate node. The inclination and orientation of the trifoliate leaves serve as critical indicators of developmental progress and maturity (Grassini et al., 2021). Studies have also detailed that the unfolding and horizontal orientation of the leaves are significant markers in identifying the completion of specific growth stages (Herrero-Huerta et al., 2020).

We observed that the general labeling method and the proposed labeling method yielded different performance metrics for varieties with short internodes. The general labeling model achieved a precision of 89.92%, recall of 69.89%, accuracy of 85.93%, and an F1 score of 0.7865. In contrast, the proposed labeling model attained a precision of 93.36%, recall of 74.58%, accuracy of 88.56%, and an F1 score of 0.8292. For varieties with short internodes, the proposed labeling method demonstrated improvements, with increases of 3.44% in precision, 4.69% in recall, 2.93% in accuracy, and 0.0427 in the F1 score.

However, for varieties with long internodes, the general labeling method exhibited superior values with an increase in precision of 4.42%, recall of 2.63%, accuracy of 1.47%, and an F1 score of 0.0346 (**Table 3**). Both the general labeling method and proposed labeling methods effectively detected leaf growth stages from the 1st to the 5th leaf during the soybean growth process. In varieties with long internodes, where internodes are spacious and leaf overlap is minimal, the general labeling method (**Figure 12a**) successfully detected all lobes. However, the proposed labeling method (**Figure 12b**) encountered false detections starting from the third lobe. This discrepancy is due to the long internode and lack of overlapping features. The general labeling method’s performance improved with wider leaf internodes, peaking in varieties with long internodes, while the proposed labeling method performed best in mid-nodal varieties. Understanding the precise sequence of leaf development in soybeans enables us to more accurately predict growth stages, which is crucial for proactive planning and resource allocation (Shi et al., 2020). This precision allows for optimized irrigation and fertilizer use, ensuring that plants receive essential nutrients at critical growth phases (Shi et al., 2020; Chaffai et al., 2024).

Moreover, knowledge of the timing for specific leaf stages aids in selecting appropriate planting dates tailored to various regions and seasons. Additionally, any deviations from the expected leaf development can indicate plant stress, prompting early interventions to address nutrient deficiencies, pests, or diseases, ultimately reducing potential yield losses (Parkash and Singh, 2020; Zhou et al., 2020; Lu et al., 2021). Our investigation indicates a potential correlation between the timing of specific leaf stages and the final yield of soybeans. Utilizing data on foliate sequence enables the development of more accurate models for predicting yield, integrating crucial information about leaf development. This approach also supports the creation of robust simulations that forecast soybean growth and yield under diverse environmental conditions. By continuously monitoring foliate sequences in real-time, farmers can implement more precise and efficient agricultural practices. This allows us for tailored resource allocation, enabling targeted applications of pesticides or fungicides as needed. This approach not only minimizes waste but also reduces environmental impact, demonstrating a proactive approach to sustainable farming practices.

## Conclusion

This research utilized artificial intelligence to automate the extraction, recording, and analysis of soybean growth data. To enhance the accuracy of growth analysis based on labeling methods, learning was conducted using an alternative labeling approach, considering both the general labeling method and soybean characteristics. Both the general and proposed labeling methods successfully detected compound leaves for soybean growth analysis. The general labeling method excelled in varieties with long internodes where compound leaves were distinct and independent, while the proposed labeling method outperformed in varieties with intermediate growth habit with narrow leaf internodes and extensive leaf overlap. Depending on the characteristics of the target object, superior performance can be achieved by selecting either a general labeling method for clear and independent labeling or a method that incorporates surrounding context. This approach facilitates automated analysis of soybean growth in large-scale testing systems. While the proposed labeling strategy has shown promising results in enhancing leaf detection performance, several avenues remain for future exploration. First, considering the substantial manual effort involved in generating labeled datasets, future studies could investigate semi-automated annotation approaches, such as active learning, human-in-the-loop systems, or self-supervised pretraining, to reduce annotation burden while maintaining labeling precision. Second, integrating leaf landmark detection or instance segmentation techniques would facilitate more accurate tracking of individual leaves over time, thereby enabling more precise growth stage classification and disease localization. Third, while this study was conducted under controlled indoor conditions, extending the framework to real-world field environments—with varying lighting, occlusion, and plant stress conditions—will be critical for practical deployment. Finally, the incorporation of multi-modal data (thermal, hyperspectral, or 3D LiDAR data) in combination with deep learning models may provide deeper physiological insights and improve model robustness under diverse phenotypic expressions. These directions will not only improve the generalizability of soybean phenotyping systems but also contribute to building more scalable, intelligent crop monitoring solutions for precision agriculture.

## Data Availability

The original contributions presented in the study are included in the article/supplementary material. Further inquiries can be directed to the corresponding authors.
